# Adult-onset primary Ewing’s sarcoma of the right atrium: a case report

**DOI:** 10.1186/s40792-019-0727-1

**Published:** 2019-11-06

**Authors:** Jun Ushigusa, Yosuke Mukae, Masanori Takamatsu, Eijiro Nogami, Akira Furutachi, Manabu Itoh, Junji Yunoki, Takahiro Nishida

**Affiliations:** 1 Center for Graduate Medical Education Development and Research, Saga University Hospital, Saga, Japan; 2 Department of Thoracic and Cardiovascular Surgery, Faculty of Medicine, Saga University, 5-1-1, Nabeshima, Saga City, Saga 849-8501 Japan

**Keywords:** Cardiac Ewing’s sarcoma, Right atrium, Adult-onset

## Abstract

**Background:**

Primary cardiac tumors, which are only detected in 0.001–0.03% of autopsies, are rare. Only 25% of primary cardiac tumors are malignant, of which 95% are sarcomas. Ewing’s sarcoma, one of the Ewing’s sarcoma of family tumors, is thought to be derived from neural crest cells. While Ewing’s sarcoma usually presents in the bone of children, Ewing’s sarcoma of cardiac origin is rare, with only a few reports described in the literature. The prognosis is unpredictable because of the scarcity and unestablished treatment. We herein report an extremely rare case of primary cardiac Ewing’s sarcoma in the right atrium of a 64-year-old man.

**Case presentation:**

The patient is a 64-year-old Japanese male who was referred to our hospital to treat a floating mass of the right atrium (RA). Although the patient was asymptomatic, we performed an operation to urgently resect the floating mass on the next day of admission due to the risk of pulmonary embolism. The operation was performed under cardiopulmonary bypass and cardiac arrest. We resected the tumor with at least 1.5 cm of the RA wall as a margin. The postoperative pathological diagnosis of the mass was compatible with a primitive neuroectodermal tumor (PNET, a form of Ewing’s sarcoma). The cells were positive for CD56, CD99, and Vimentin and negative for S-100 and Desmin. Although no malignant cells were observed in the margin of the resected RA wall and the sarcoma was completely resected, he was transferred to another hospital to receive adjuvant postoperative chemotherapy to improve the prognosis by preventing subclinical micrometastasis.

**Conclusions:**

We experienced an extremely rare case of primary cardiac Ewing’s sarcoma in the right atrium of a 64-year-old man, which was successfully resected under cardiac arrest. Although the sarcoma was completely resected, postoperative chemotherapy and long-term follow-up are recommended for patients with primary cardiac sarcoma because of the high rates of metastasis and recurrence.

## Background

Primary cardiac tumors, which are only detected in 0.001–0.03% of autopsies, are rare. Only 25% of primary cardiac tumors are malignant, of which 95% are sarcomas. The most frequent cardiac sarcomas are hemangiosarcoma and rhabdomyosarcoma.

The Ewing’s sarcoma of family tumors, which includes Ewing’s sarcoma, primitive neuroectodermal tumor (PNET), neuroepithelioma, and skin tumor, is thought to be derived from neural crest cells. While Ewing’s sarcoma presents in the bone of children, Ewing’s sarcoma of cardiac origin is rare, with only a few reports described in the literature. The prognosis is unfavorable.

We herein report an extremely rare case of primary cardiac Ewing’s sarcoma in the right atrium of a 64-year-old man.

## Case report

**Fig. 1:**
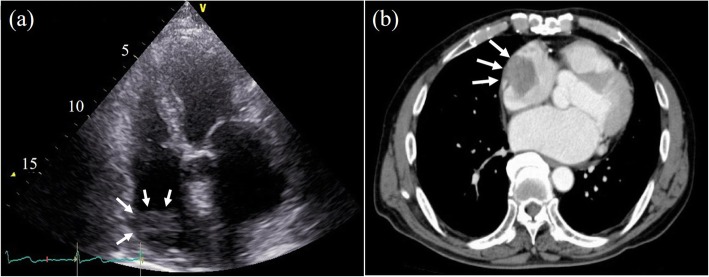
**a** A 45 × 27 mm mass (white arrows) was observed as an area of low density near the right atrial free wall on preoperative contrast-enhanced computed tomography. **b** A round floating mass (white arrows) with a relative low echogenicity was recognized in the right atrium in the preoperative four-chamber view of transthoracic echocardiography

A 64-year-old Japanese male patient was referred to our hospital to treat a floating mass of the right atrium (RA). He had undergone resection of sigmoid cancer 5 years previously and endoscopic submucosal dissection for early carcinoma of the esophagus 5 years ago. Follow-up computed tomography (CT) revealed a mass of 45 × 27 mm in the RA with a low-density area (Fig. 1a). Preoperative transthoracic echocardiography revealed a round mass with low echo density on the free wall of the RA (Fig. 1b), which was suspected to represent thrombosis, cardiac myxoma, or a relapsed metastatic tumor. Although the patient was asymptomatic, we planned to urgently resect the floating mass on the next day of admission due to the risk of pulmonary embolism.

The operation was performed under cardiopulmonary bypass. Normothermic cardiopulmonary bypass was established by using an arterial cannula to the ascending aorta and two venous cannulas into the superior vena cava and inferior vena cava with a left ventricular venting cannula through the right upper pulmonary vein. The ascending aorta was cross-clamped, and the heart was arrested with cold blood cardioplegia.

**Fig. 2:**
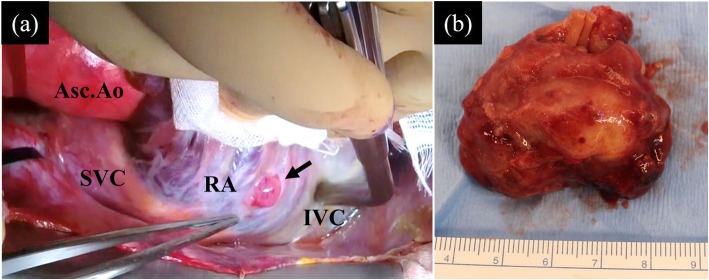
**a** A small part of the mass (black arrow) was identified protruding though the right atrial free wall. **b** The 45-mm mass was soft and elastic with a smooth surface. Asc.Ao, ascending aorta; SVC, superior vena cava; RA, right atrium; IVC, inferior vena cava

We observed a large, pedunculated, relative tense mass of 45 mm × 27 mm in size on the free wall of the RA. We could directly identify a small part of the mass protruding though the RA wall, which was connected to the RA wall (Fig. 2a). We resected the tumor with at least 1.5 cm of the RA wall as a margin (Fig. 2b). We added tricuspid annuloplasty with Carpenter Edwards Physio-ring II of 28 mm (Edwards Lifesciences Corporation, CA, USA) for tricuspid valve regurgitation, and the left atrial appendage was closed with a 40-mm AtriClip (AtriCure, Inc., Mason, OH, USA) and bilateral pulmonary vein isolation by an AtriCure (AtriCure, Inc.) to prevent thrombosis formation due to chronic atrial fibrillation. The operation time, cardiopulmonary bypass time, and aorta cross-clamp time were 317 min, 145 min, and 88 min, respectively. The patient recovered uneventfully and returned to home on foot on the 13th day after the operation.

**Fig. 3:**
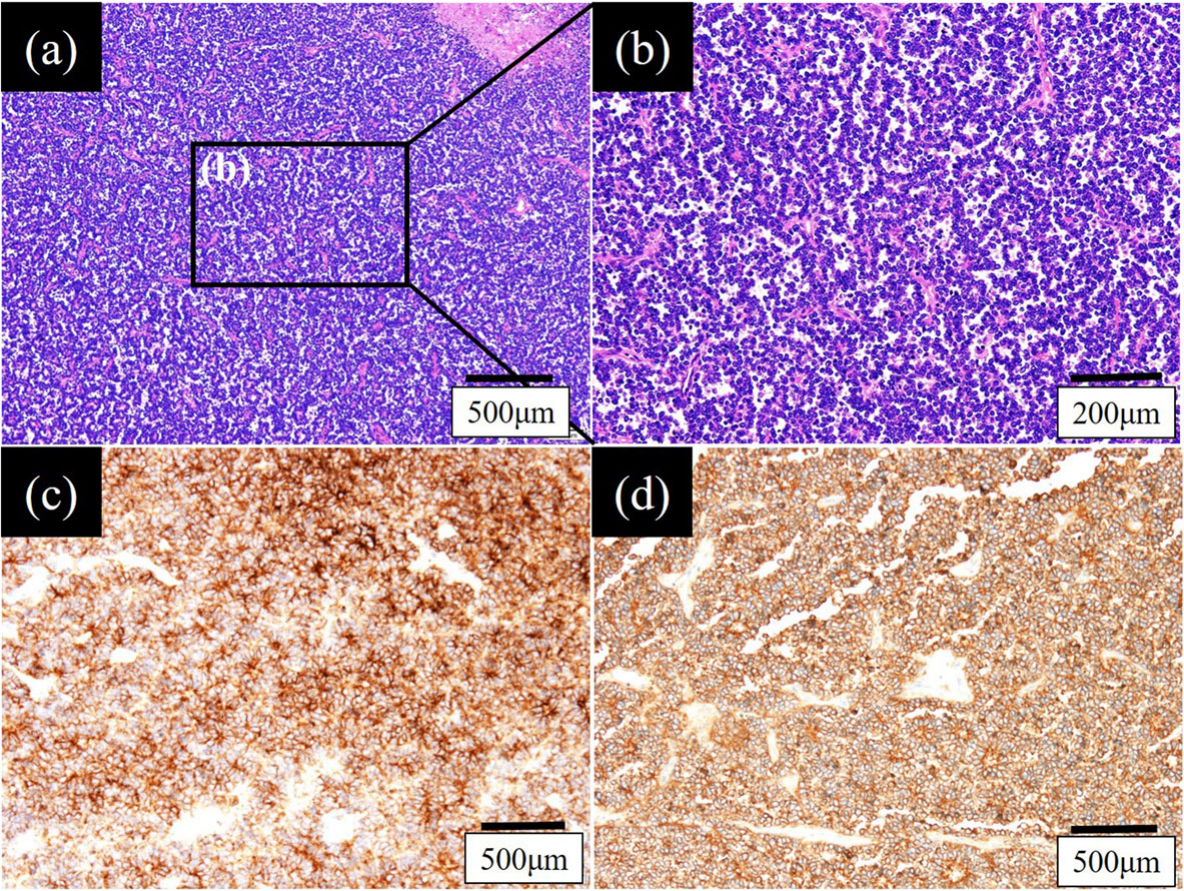
Hematoxylin and eosin staining revealed that atypical small cells with oval nuclei had multiplied into a micropapillary form with a partially rosette formation (**a**, **b**). These cells were positive for CD56 (**c**) and CD99 (**d**)

The postoperative pathological diagnosis of the mass was compatible with a primitive neuroectodermal tumor (PNET), a form of Ewing’s sarcoma. Atypical cells with small oval nuclei propagated into a micropapillary form with a partial rosette formation (Fig. 3a, b); some parts contained necrotized tissue. These cells were positive for CD56 (Fig. 3c), CD99 (Fig. 3d), and Vimentin and negative for S-100 and Desmin. No malignant cells were observed in the margin of the resected RA wall. Although the sarcoma was completely resected, he was transferred to another hospital to undergo adjuvant postoperative chemotherapy and radiotherapy to improve the prognosis by preventing subclinical micrometastasis. The final diagnosis was primary cardiac Ewing’s sarcoma because no primary lesions were observed in other organs on fludeoxyglucose-positron emission tomography (FDG-PET) or bone scintigraphy, which was performed in another hospital. As the adjuvant postoperative chemotherapy protocol, he received VDC-IE therapy as follows: VDC with vincristine 1.4 mg/m^2^ (d1), doxorubicin 37.5 mg/m^2^ (d1–2), and cyclophosphamide 1200 mg/m^2^ (1d); and IE with ifosfamide 1.8 g/m^2^ (d1–5) and etoposide 100 mg/m^2^ (d1–5). VDC-IE therapy was the same as that for born-derived Ewing’s sarcoma. He had experienced no recurrence as of 2 months after the operation.

## Discussion

We experienced a case of a primary cardiac tumor that occurred on the RA wall, which was pathologically diagnosed as Ewing’s sarcoma. Primary sarcoma of the bone is reported to develop 0.0001–0.03% of people, and cardiac primary sarcoma is much rarer than benign cardiac tumors. Among the primary cardiac sarcomas, Ewing’s sarcoma is quite rare. To our knowledge, only a few reports have described primary cardiac Ewing’s sarcoma [1, 2] and no reports have described primary Ewing’s sarcoma of the RA wall.

The pathological findings of the mass of our patient were compatible with PNET, a form of Ewing’s sarcoma, based on the identification of atypical small cells with oval nuclei which had propagated into a micropapillary form with partial rosette formation; some parts contained necrotized tissue. The specimen was positive for CD56, CD99, and Vimentin. When we make a diagnosis of “primary” cardiac sarcoma, we should evaluate the bone as well as the chest wall, because primary sarcoma usually occurs in the long bones of the lower extremities and the chest wall, although such sarcomas rarely metastasize to the heart or lung [3]. FDG-PET or bone scintigraphy are the most appropriate examinations to rule out metastatic cardiac sarcoma. In the present case, we diagnosed the tumor as “primary” because no primary lesions were observed in other organs on FDG-PET or bone scintigraphy performed in another hospital.

In general, when a cardiac mass is found, vegetation due to infective endocarditis, thrombosis, or a cardiac tumor (including myxoma) is suspected. Three fourths of cardiac myxomas arise in the left atrium and show a low-density area on enhanced CT; sometimes the patient presents fever due to interleukin-6. In contrast, malignant cardiac tumors appear as a high-density area on enhanced CT because they normally have abundant vessels with rich blood perfusion. However, some malignant cardiac tumors might show a low-density area in the middle of the mass if the blood perfusion of the tumor is poor. In fact, in our case, the RA mass appeared as a low-density area on enhanced CT because the pathological findings revealed poor blood perfusion, in spite of the formation of some vessels. It should be noted that more than 80% of right-sided sarcomas were found to be metastatic tumors at the time of detection [4], which leads to a poor prognosis. Thus, we should carefully deal with right-sided heart tumors as a suspected malignant tumor and not leave malignant cells during surgery. Unlike sarcoma of other organs, the resection margin of cardiac sarcoma depends on its location in the heart and the native cardiac chamber size. The tumor was located at the free wall of the RA, which was why we were able to resect the tumor with a sufficient margin without any complications, such as atrioventricular block or heart failure. Therefore, he is alive with no recurrence.

The common causes of death in the acute phase of cardiac sarcoma are pulmonary embolism (PE) and cardiac tamponade caused by metastatic pericardium [4]. In general, we need to resect a cardiac mass urgently, regardless of whether it is a pathological malignancy, because the cardiac masses seem to be associated with an increased risk of embolism. Surgical resection with wide margin is a gold standard treatment of cardiac mass. The survival rate of patients with malignant cardiac tumors is significantly higher among patients who undergo complete resection [5]. In addition, if we have enough time before the operation, magnetic resonance imaging (MRI) is recommended in order to understand the anatomic features [6], as this may enable more secure complete resection.

Surgical cardiac resection, especially with the RA wall around the sinus node, might have a negative influence on the cardiac conduction system and, depending on the part that is excised, might necessitate the implantation of a permanent cardiac pacemaker. In the present case, the mass existed on the free wall of the RA and the surgical procedure did not cause any arrhythmia, including sick sinus syndrome or atrioventricular block.

There are no clear definite treatments for cardiac Ewing’s sarcoma because of the small number of patients and the poor prognosis. Llombart-Cussac et al. [5] reported that the 2-year survival rate of patients with primary cardiac sarcoma was only 26%. Thus, postoperative chemotherapy is recommended for patients who undergo surgical resection of cardiac sarcoma, even if the margin of the resected tissue is pathologically negative and there is no apparent metastasis [4]. Some reports have shown that doxorubicin treatment [7] is associated with improved survival after the resection of cardiac sarcoma. However, anthracycline drugs, including doxorubicin, increase cardiac toxicity. Radiotherapy is administered in limited cases to patients who show poor tolerance of chemotherapy or as palliative therapy [8, 9]. After surgery, the efficient treatment of cardiac sarcoma—including Ewing’s sarcoma—requires a multidisciplinary approach from cardiologists, oncologists, and radiologists.

## Conclusion

We reported the first case of an adult-onset primary cardiac Ewing’s sarcoma of the right atrium, which was successfully resected under cardiac arrest. Appropriate postoperative chemotherapy and long-term follow-up are recommended for patients with primary cardiac sarcoma because of the high rates of metastasis and recurrence.

## Data Availability

Data sharing not applicable to this article as no datasets were generated or analyzed during the current study.

## Abbreviations

CT: Computed tomography
FDG-PET: Fluorodeoxyglucose-positron emission tomography
MRI: Magnetic resonance imaging
PE: Pulmonary embolism
PNET: Primitive neuroectodermal tumor
RA: Right atrium
